# Reviving the poppy seed test for the diagnosis of colovesical fistula: A case report from a single rural center experience

**DOI:** 10.1002/ccr3.9500

**Published:** 2024-10-21

**Authors:** Khang Duy Ricky Le, Shasha Haycock, Annie Jiao Wang, Leslie Yeaman

**Affiliations:** ^1^ Department of General Surgical Specialties The Royal Melbourne Hospital Melbourne Victoria Australia; ^2^ Department of General Surgery Northeast Health Wangaratta Wangaratta Victoria Australia; ^3^ Geelong Clinical School Deakin University Geelong Victoria Australia; ^4^ Faculty of Medicine, Dentistry and Health Sciences The University of Melbourne Melbourne Victoria Australia; ^5^ Department of Urology Northeast Health Wangaratta Wangaratta Victoria Australia

**Keywords:** colonoscopy, colovesical fistula, cystoscopy, enterovesical fistula, poppy seed test

## Abstract

**Key Clinical Message:**

The poppy seed test is a valuable, cost‐effective, and sensitive initial diagnostic investigation to identify the presence of colovesical fistula, particularly for clinicians in remote or rural areas with lack of access to specialist radiologic, endoscopic, surgical, or urological support. The poppy seed test therefore may act as a bridge toward the commencement of appropriate referral pathways for the management of colovesical fistula.

**Abstract:**

The diagnosis of colovesical fistula is resource intensive, often requiring a combination of radiological investigation and endoscopy. The poppy seed test is a non‐invasive and cost‐effective alternative that has been demonstrated to identify presence of colovesical fistula with high sensitivity. There is however a noticeable paucity of recommendations for the poppy seed test in the diagnostic approach to colovesical fistula due to significant advancements in alternative radiologic and endoscopic technologies. Despite this, in resource‐challenged areas with lack of specialist support, the poppy seed test is a cost‐effective, accessible and safe test that can be performed to confirm the presence of colovesical fistula. We report a case of a 79‐year‐old man with a past history of colorectal cancer managed surgically with adjuvant chemoradiotherapy who develops noisy micturition, pneumaturia, and fecaluria 22 years after treatment. Given the suspicion of colovesical fistula, computed tomography imaging and cystoscopy were performed which was unable to demonstrate an overt fistula. The poppy seed test was utilized and demonstrated presence of poppy seeds in the urine, confirming the presence of a fistula. The patient was referred to a specialist surgical center for consideration of further management. The poppy seed test is a valuable initial diagnostic investigation for clinicians in remote or rural areas with lack of access to specialist radiologic, endoscopic, surgical, or urological support in confirming the presence of a colovesical fistula and initiating referral pathways for the management of this condition.

## 1 BACKGROUND

The diagnosis of colovesical fistula is often challenging, resource‐intensive, and expensive. Ingestion of poppy seeds has been described in the literature as a cost‐effective initial diagnostic test in patients suspected to have either an enterovesical fistula or a colovesical fistula with high detection rate and sensitivity.^1^ The test involves ingestion of poppy seeds and visual inspection of the urine for the presence of seeds within a 48‐h period.^2^ The presence of poppy seeds in the urine confirms the presence of fistula. In rural centers with no immediate endoscopic or urological support, the poppy seed test is a practical and inexpensive first step to workup patients with suspected colovesical fistula. We present a case of a 79‐year‐old man who, despite negative conventional diagnostic investigations, was proven to have a colovesical fistula with the poppy seed test.

## 2 CASE PRESENTATION

The patient provided written consent for the de‐identification and use of their medical information and data for the generation and publication of this case report.

### 2.1 Case history and examination

A 79‐year‐old man was referred to our rural surgical service following complaints of 4 weeks of noisy micturition, pneumaturia, and fecaluria. Further enquiry was unremarkable for features of infection including dysuria, fever, and rigors. These findings occur on the background of colorectal cancer in which the patient underwent an ultra‐low anterior resection with formation of a covering loop ileostomy in 2002, followed by 6 months of adjuvant chemotherapy and radiotherapy. He subsequently underwent stoma reversal following remission of colorectal cancer and continues on regular yearly colonoscopic surveillance. The last colonoscopy in October 2023 demonstrated no evidence of recurrence; however, there were features of radiation colitis noted around the patent colorectal anastomosis and the rectum. His past medical history is otherwise significant for right coronary artery atherosclerosis with a positive stress test leading to percutaneous insertion of a drug‐eluting stent in 2016, hypercholesterolemia, hypertension and previous episodes of adhesional small bowel obstruction.

### 2.2 Differential diagnosis, investigations, and management

**FIGURE 1:**
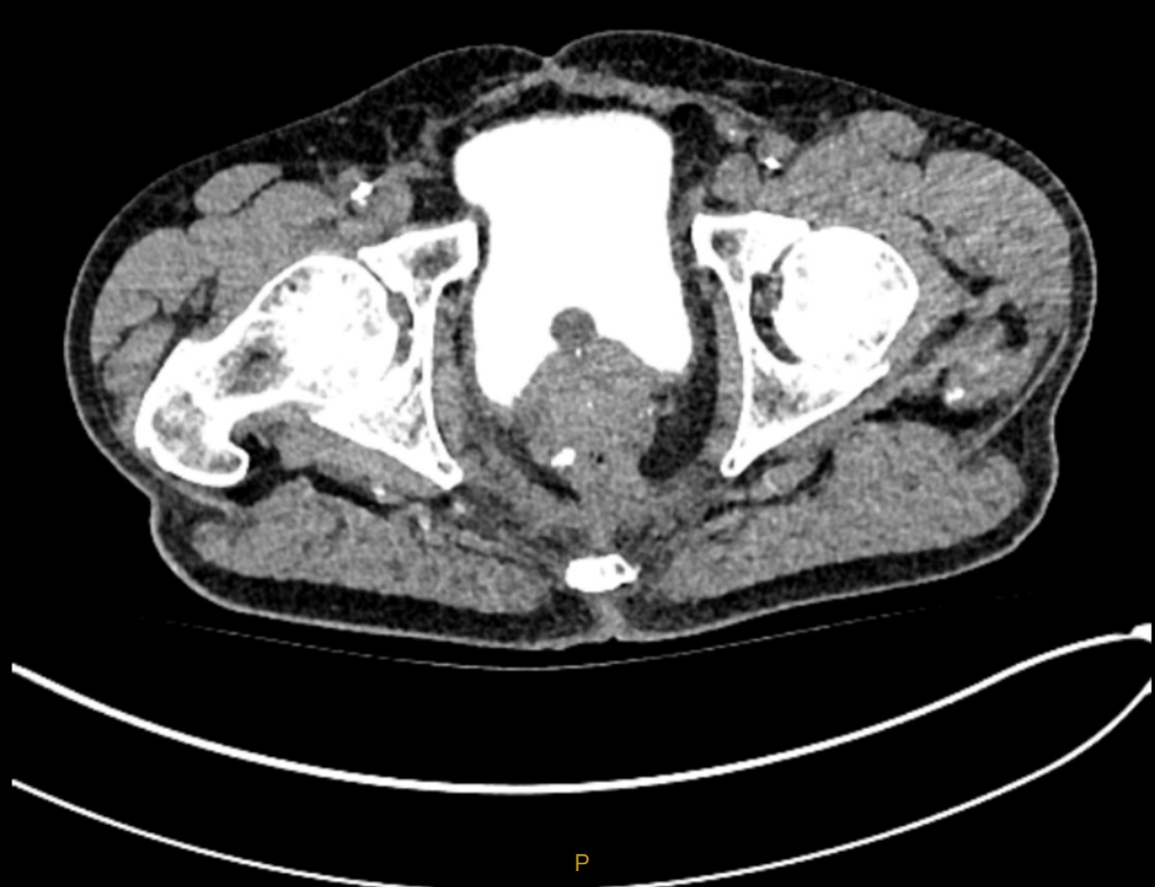
Computer tomography cystogram demonstrating absence of extra‐luminal bladder contrast consistent with a negative finding for colovesical fistula.

**FIGURE 2:**
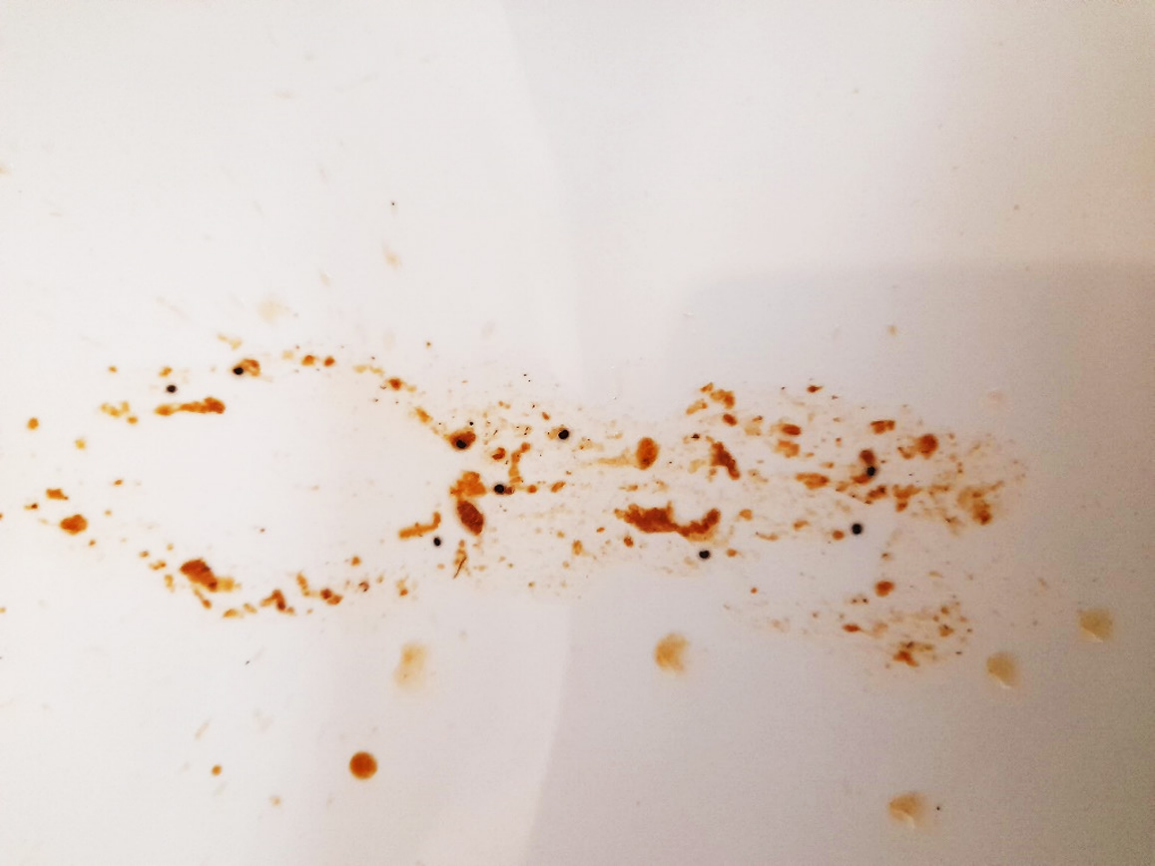
Clinical photograph of urine sample demonstrating presence of fecaluria and presence of poppy seeds.

Given the suspicion of a colovesical fistula, a computed tomography (CT) cystogram was performed which was unremarkable (Figure 1). He subsequently underwent a flexible cystoscopy which similarly was unable to identify an overt fistula, nor any bladder lesions. Despite this, cystoscopy demonstrated suspicious findings for an unidentified colovesical fistula including non‐specific cystitis of predominantly the posterior bladder region including the trigone and posterior bladder neck region in addition to presence of free air in the same region. The patient was advised to perform the poppy seed test where he consumed approximately 50 mg of poppy seeds and collected his urine in the following period of 24–48 h. This test was significant for urine that contained poppy seeds and therefore strongly suggesting the presence of a colovesical fistula (Figure 2). The patient was subsequently referred for repeat colonoscopy and consideration of bowel resection for the management of this condition.

## 3 DISCUSSION

Colovesical fistula commonly present in older males (Male: 85.7%, Female: 14.3%) with median age of 66 years.^3^ The majority of these patients present with urinary symptoms including dysuria (60%), fecaluria (35%–51%), pneumaturia (35%–71.4%), and recurrent urinary tract infections (67%), with or without abdominal pain. These symptoms occur on a background of risk factors including history of diverticulitis, colon cancer, invasive bladder cancer, Crohn's disease, and brachytherapy for prostate or genitourinary cancers.^1^, ^3^ Of these symptoms, fecaluria and pneumaturia are considered pathognomonic.^3^ Although these classic features are seen in our present case study, a recent systematic review demonstrated they are only present in up to half of all cases.^4^ In addition, non‐specific symptoms such as altered bowel habits may be the only sign of this condition. Given this, definitive diagnosis of colovesical fistula often requires months of intensive monitoring.^5^ The diagnostic approach for investigation of colovesical fistula predominantly relies on initial radiographical investigations with barium enema, cystography, CT imaging with rectal or bladder contrast, or magnetic resonance imaging (MRI) of the pelvis with seldomly used adjuncts such as ingestion of oral charcoal or dye. Endoscopic investigation with cystoscopy and colonoscopy are particularly useful in cases of non‐diagnostic radiological investigation to directly visualize the site of the fistula. Endoscopy also facilitates decision‐making regarding operative management of the identified fistula. Furthermore, biochemical tests such as with the Bourne test or nuclear imaging with 51‐chromium nuclear scans have also been suggested; however, their use is poorly reported in contemporary workup. The poppy seed test is another method by which fistula can be confirmed, however has fallen out of favor in light of technological advancement in radiologic and endoscopic technology. Most importantly, generally patients require several studies prior to the diagnosis of colovesical fistula. This has multiple implications including higher healthcare costs, delays to treatment and psychological impact on patients as a result of uncertainty and anxiety.^3^ Moreover, rural and remote areas may not have timely access to endoscopic and urologic support to perform these investigations, and in some areas, cross‐sectional imaging through CT or MRI is limited. The poppy seed test provides a cost‐effective and reasonable alternative and bridge for the diagnostic workup of colovesical fistula, particularly in these settings. Furthermore, the poppy seed test also provides a reasonable and sensitive approach to testing for fistula in patients where there is minor suspicion but without overt signs of pneumaturia or faecaluria.

At present, there is a noticeable paucity in evidence evaluating the poppy seed test in contemporary pathways of diagnosis for colovesical fistula. A literature search performed in June 2024 of Medline and Pubmed databases identified only three full‐text peer reviewed, articles, including two prospective studies and one retrospective study (*n* = 11, 20 and 49, respectively). Despite being single center trials of small sample size, the authors of these studies report benefits of increased sensitivity and cost‐effectiveness of the poppy seed test compared to conventional investigations. When assessed against surgically demonstrated fistula, the poppy seed test had a 94.6%–100% detection rate for colovesical fistula.^1^, ^2^, ^3^ Of note, for the two patients with surgically demonstrated fistula and a negative poppy seed test, endoscopic and imaging studies were also negative.^3^ The poppy seed test also demonstrates the improved sensitivity compared to alternative studies including CT (27%–70%), colonoscopy (0%–8.5%), 51‐chromium (80%), cystoscopy (10.2%–50%), barium enema (25.7%–43%), cystogram (9%–20%), and MRI (60%).^1^, ^2^, ^3^ The test is also the most inexpensive ($5.37 per study) compared to 51‐chromium nuclear scans (USD490.83) and CT (USD652.92).^1^ Additional benefits compared to alternative methods of fistula detection include improved taste compared to charcoal and lack of radiation.^2^ This highlights the value of the poppy seed test as an affordable and sensitive initial diagnostic tool. Despite this, limitations of the poppy seed test include the inability of this test to delineate the anatomy of the fistula, a key consideration when it comes to operative management. Thus, the less sensitive investigations of radiography with barium enema, cystoscopy, and CT remain valuable for surgical planning and guiding management following initial confirmation. Nonetheless, our case reports identified a unique niche for the poppy seed test in rural or remote settings that lack endoscopic and urology services, offering clinicians an efficient means to work up colovesical fistula while coordinating referral pathways.

## AUTHOR CONTRIBUTIONS

**Khang Duy Ricky Le:** Conceptualization; data curation; formal analysis; investigation; methodology; project administration; supervision; validation; writing – original draft; writing – review and editing. **Shasha Haycock:** Data curation; formal analysis; investigation; methodology; resources; validation; writing – original draft; writing – review and editing. **Annie Jiao Wang:** Data curation; formal analysis; investigation; methodology; resources; validation; writing – original draft; writing – review and editing. **Leslie Yeaman:** Formal analysis; supervision; validation; writing – review and editing.

## FUNDING INFORMATION

Nil.

## CONFLICT OF INTEREST STATEMENT

Nil.

## ETHICS STATEMENT

The case report generation process was discussed with our local ethics and governance team. No formal ethics approval was required following the discussions and therefore was waived.

## CONSENT

Written informed consent was obtained from the patient to publish this report in accordance with the journal's patient consent policy.

## CONSENT TO PARTICIPATE

The patient provided consent for the de‐identification and use of their medical information and data for the generation and publication of this case report.

## Data Availability

Data can be requested from corresponding author when required. All relevant data has been provided in the generation of this manuscript which is intended for open access publication.

## References

[1] Kwon EO , Armenakas NA , Scharf SC , Panagopoulos G , Fracchia JA . The poppy seed test for colovesical fistula: big bang, little bucks! J Urol. 2008;179(4):1425‐1427. 18289575 10.1016/j.juro.2007.11.085

[2] Schwaibold HPC , Geist E , Hartung R . Oral intake of poppy seed: a reliable and simple method for diagnosing vesico‐enteric fistula. J Urol. 2001;166(2):530‐531. 11458060 10.1016/s0022-5347(05)65976-9

[3] Melchior S , Cudovic D , Jones J , Thomas C , Gillitzer R , Thüroff J . Diagnosis and surgical management of colovesical fistulas due to sigmoid diverticulitis. J Urol. 2009;182(3):978‐982. 19616793 10.1016/j.juro.2009.05.022

[4] Zizzo M , Tumiati D , Bassi MC , et al. Management of colovesical fistula: a systematic review. Minerva Urol Nephrol. 2022;74(4):400‐408. 34791866 10.23736/S2724-6051.21.04750-9

[5] Scozzari G , Arezzo A , Morino M . Enterovesical fistulas: diagnosis and management. Tech Coloproctol. 2010;14(4):293‐300. 20617353 10.1007/s10151-010-0602-3

